# Supplementary material for: A BPTF Inhibitor That Interferes with the Multidrug Resistance Pump to Sensitize Murine Triple-Negative Breast Cancer Cells to Chemotherapy
Source: Int J Mol Sci. 2024 Oct 22;25(21):11346. doi: 10.3390/ijms252111346 (PMC11545213; doi:10.3390/ijms252111346)
Supplement: Supplementary file 1 [file ijms-25-11346-s001.zip › 7_25_24 supplmentalrevisions.pdf]

Supplemental Figures

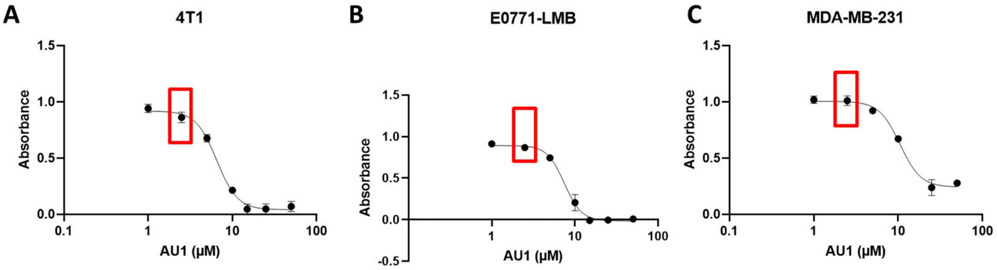

Supplemental Figure S1. Sensitivity of 4T1, E0771-LMB, and MDA-MB-231 cells to AU1. Dose-response curves were generated with 4T1 (A), E0771-LMB, and MDA-MB-231 (C) cells. For all cell lines, 2.5 µM AU1 (boxed data point) was selected as the designated concentration for subsequent experiments. Cells were exposed to serially diluted AU1 for 96 h prior to evaluation with the MTS viability assay. Results are means ± SEM of at least three independent experiments.

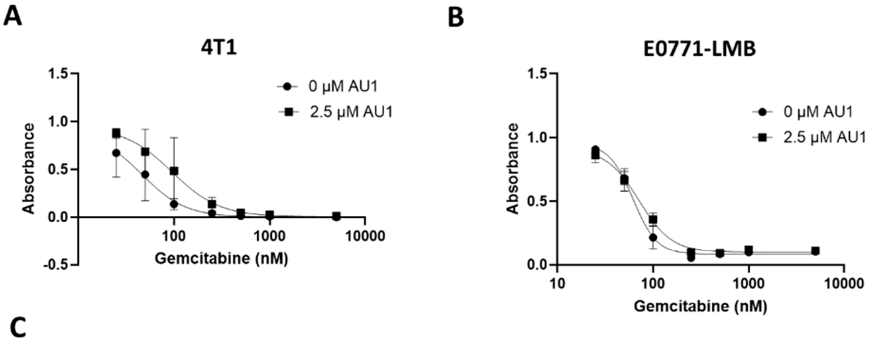

C

IC_{50} (µM)	0 µM AU1	2.5 µM AU1	Ctrl /AU1
4T1	46.0 ± 47.5	95.3 ± 46.0	0.483
E0771-LMB	61.9 ± 4.4	71.7 ± 9.6	0.863

Supplemental Figure S2. Dose-response of 4T1 cells treated with gemcitabine with and without AU1 in combination. Dose-response curves of gemcitabine with and without AU1 were generated with (A) 4T1 and (B) E0771-LMB cells. Corresponding IC_{50} values and calculated fold-change are indicated in the inset table (C). Plated 4T1 cells were allowed to adhere to 96-well plates, pretreated with AU1 overnight as appropriate, and treated the following day with serially diluted chemotherapies with and without AU1 for 96 h. Cells were evaluated via the MTS viability assay. Results are means ± SEM of at least three independent experiments.

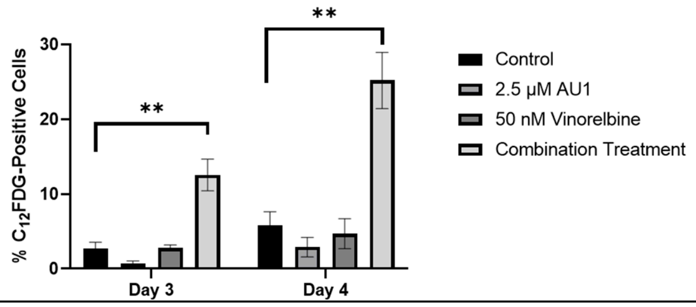

Supplemental Figure S3. Quantification of senescence in 4T1 cells treated with vinorelbine and AU1 alone or in combination. Senescence was measured using C_{12}FDG, a fluorogenic probe for β-galactosidase, with flow cytometry, as previously described ([51]). β-galactosidase-positive cells were quantified for individual treatments and combination treatments of 2.5 µM AU1 and 50 nM vinorelbine on days 3 and 4. **p ≤ 0.01 and ns compared to controls by one-way ANOVA with Tukey post-hoc. Results are means ± SEM of at least three independent experiments.

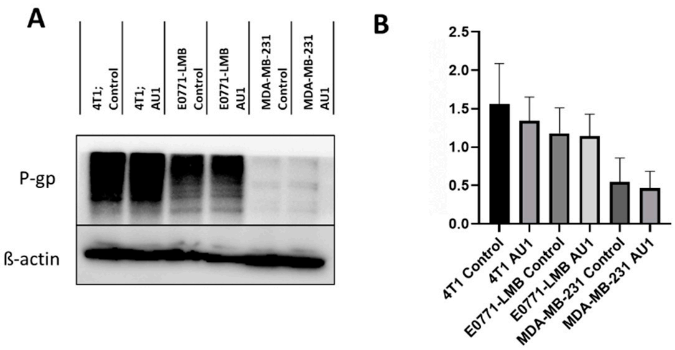

Supplemental Figure S4. Western blotting with and without AU1 administration. Western blot of (A) P-gp in 4T1, E0771-LMB, and MDA-MB-231 cells with and without AU1 administration as well as (B) corresponding western quantification Cells were plated and treated with AU1 for 96 h, as appropriate. All blots are representative results taken from 3 biological replicates. Quantification results are means ± SEM of at least three independent experiments.

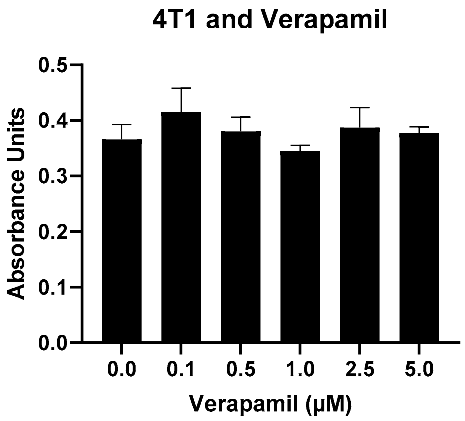

Supplemental Figure S5. Influence of verapamil on viability of 4T1 cells. Plated 4T1 cells were allowed to adhere to 96-well plates and treated the following day with serially diluted verapamil for 96 h. Cells were evaluated via the MTS viability assay. Results are means ± SEM of at least three independent experiments.

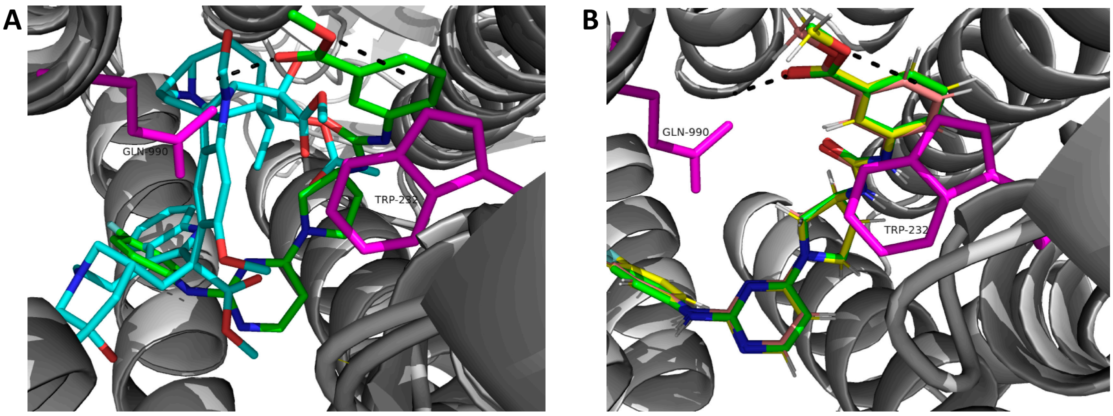

Supplemental Figure S6. Comparison of AU1 (green sticks) to the Cryo-EM (PDBID: 7A69) bound ligand vincristine (cyan sticks) in the binding pocket of MDRP. The direct hydrogen bond forming residues are shown in magenta sticks (A). Top 3 posses of AU1 from the docking experiment are shown in different colored stick representation (B).
